# The Akcaalan Mortality Score: A Novel Mortality Score to Predict 3-Year Mortality for Elderly Hip Fractures

**DOI:** 10.3390/jcm14103538

**Published:** 2025-05-18

**Authors:** Serhat Akcaalan, Batuhan Akbulut, Kemal Memis, Ceyhun Caglar, Mahmut Ugurlu, Mehmet Ismail Safa Kapicioglu, Metin Dogan

**Affiliations:** 1 Orthopedics and Traumatology Department, Ankara City Hospital, 06800 Çankaya, Turkey; drbatuhanakbulut@gmail.com (B.A.); kemalm40@gmail.com (K.M.); ceyhun.caglar@hotmail.com (C.C.); mugurlu@gmail.com (M.U.); safa.kapicioglu@gmail.com (M.I.S.K.); drmetindogan@gmail.com (M.D.); 2 Orthopedics and Traumatology Department, Ankara Yildirim Beyazıt University, 06760 Çubuk, Turkey

**Keywords:** elderly, hip, fracture, mortality, score

## Abstract

**Backround/Objectives**: This study aimed to create a scoring system that can predict the mortality for hip fractures in the elderly, which have high mortality and morbidity rates, by using blood parameters and demographic data at admission. **Methods**: Patients admitted to the hospital due to a hip fracture between January 2016 and March 2021 were included in the study. A scoring system was created using the patient’s age and sex at first admission and hemoglobin, albumin and creatinine levels, neutrophil–lymphocyte ratio and monocyte–lymphocyte ratios. The scoring system was created by determining different cut-off values for each of these seven parameters. A total mortality score was determined for each patient using this scoring system. The 3-year follow-up for patients’ mortality during follow-up was recorded separately for each patient. Following the inclusion and exclusion criteria, the data of 1075 patients were included in the study. **Results**: All parameters listed in the methodology section were statistically significantly different between the patients who survived and those who died in the three years after hip fracture surgery (*p* = 0.0001). The total scores obtained using the mortality scoring system created by combining these parameters were also statistically significantly different between the two groups (*p* = 0.0001). If the mortality score is >11.5, the probability of the patient with a hip fracture dying within the first three years is 63.9%. **Conclusion**: The Akçaalan Mortality Score can provide predictive data for preoperative prediction to determine the 3-year mortality of elderly patients with hip fractures and may be helpful in terms of surgical timing. The name of this scoring system comes from the lastname of the corresponding author.

## 1. Introduction

Hip fractures are highly significant and challenging situations for the geriatric population [1]. By 2050, the number of global hip fracture cases is expected to surpass six million [2,3]. The most fundamental reason behind this growth over the years is the increase in life expectancy and the ratio of the elderly population in societies due to improvements in the quality of health services [4,5]. In addition to causing physical incompetency and requiring outside care, patients suffering from hip fractures create a pronounced burden on health systems and society [6,7]. It is known that hip fractures in the elderly are associated with a high mortality rate and readmission to hospital in the years immediately following the incident [8]. The mortality rate can go up as much as 13.3% within one month after a hip fracture has occurred, and the mortality rate varies between 14% and 36% within the first year [9,10].

Extensive research can be found in the literature that examines and attempts to reveal the risk factors concerning mortality after hip fractures. These studies mainly focus on the patient’s demographic characteristics, surgical style, anesthesia, postoperative management, complications, and comorbidity status of the patients [11,12,13]. Additionally, laboratory factors have been considered, and studies associated with post-hip fracture mortality abound [14,15]. Preoperative identification of the risk factors associated with high mortality and morbidity can positively affect prognosis, along with extensive evaluation and appropriate treatment methods.

Various systems are commonly utilized to anticipate hip fracture mortality in elderly patients. These systems mainly consist of parameters such as the age, sex, and the American Society of Anesthesiologists score for the patients. Apart from these, The Nottingham Hip Fracture Score and The Physiological and Operative Severity Score for the Enumeration of Morbidity and Mortality, used in 30-day mortality predictions, are two of the many scoring systems developed to predict hip fracture mortality [16,17,18]. However, there are concerns regarding the appropriate usability of these scoring systems for acute cases [19].

The parameters selected in this study, including serum albumin, creatinine, neutrophils, monocytes, and lymphocytes, were chosen based on their well-established clinical relevance and prognostic value. Serum albumin is widely recognized as an indicator of nutritional status and systemic inflammation and is frequently associated with malnutrition and cancer cachexia [20]. Creatinine serves as a sensitive marker of renal function and, indirectly, of cardiovascular sufficiency and perfusion status, particularly in elderly patients with multiple comorbidities [14]. Peripheral blood neutrophils are early markers of bacterial infection and inflammation, and elevated levels in the first blood specimen—typically obtained within a few hours after injury—may indicate pre-existing systemic infection. Monocytes contribute to chronic inflammatory responses and immune dysregulation, while lymphocytes reflect overall immune competence. Therefore, elevated neutrophil-to-lymphocyte (NLR) and monocyte-to-lymphocyte (MLR) ratios serve as surrogate indicators of a pro-inflammatory state, which has been linked with worse outcomes in various surgical and trauma populations [21].

This study aimed to determine whether the postoperative mortality ratio of a patient with a hip fracture can be predicted using evaluations conducted upon first admission. Therefore, we intended to develop a scoring system that is easy to apply at first admission and better informs interested parties about postoperative three-year mortality.

## 2. Materials and Methods

This study was designed retrospectively. The data collection process started following our hospital’s research ethics committee approval with number E1-23-4271. Patients evaluated for hip fractures between January 2016 and March 2021 were included in the study. Patients under the age of 65 at the time of admission due to hip fracture, those who had pathological fractures, subtrochanteric fractures, prior surgical history on the related hip area, multiple fractures, polytrauma patients, those previously diagnosed with or treated for malignancy, patients with isolated and greater trochanter fractures with no surgical intervention, those who had received dialysis treatment due to chronic kidney failure, and patients diagnosed with hematological diseases were excluded. Patients with active malignancies, end-stage renal disease, hematological disorders or other severe comorbidities were excluded in order to reduce the potential confounding effects of these systemic conditions on inflammatory markers. This approach aimed to provide a more homogeneous study population for accurate assessment of the proposed score’s predictive power. The laboratory parameters included in the scoring system (Hb, albumin, creatinine, NLR, and MLR) were selected based on their routine availability during the initial emergency admission process and their known association with prognosis in elderly patients. In our clinical setting, inflammatory markers, such as CRP, IL-1, IL-6, and TNF, are not included in standard admission tests, as they require additional requests and costs. For this reason, they were not incorporated into the score. Similarly, coagulation-related parameters were not considered, as patients with known bleeding disorders were excluded from the study cohort.

The blood parameters and demographic data of the patients taken upon first emergency admission were collected to develop the scoring system. However, the mortality status of the patients was obtained from the national electronic health system network. The system is 100% reliable in terms of mortality. Seven different parameters were used for the scoring. The first of these is the patient’s age when they first suffered a hip fracture. The literature clearly shows increased hip fracture mortality as age increases [22,23,24]. In a study in which the intrahospital mortality of hip fractures was evaluated, Monedero et al. grouped patients into three age groups: 65–74, 75–84, and >85, clearly revealing the relationship between this grouping and mortality [25]. Other studies have shown that hip fractures in males resulted in higher mortality [22,24]. Since various studies in the literature have indicated the effect of sex on mortality, the second parameter in our scoring system was the sex of the patient. The fracture type parameter, which is not included in these seven parameters, was excluded, as previous studies have shown that it is not associated with mortality [22,23,26,27]. Anemia is a commonly encountered problem among older people; it is associated with high mortality and morbidity rates and reduced functional status [28]. Other studies have focused on the effects of different hemoglobin (Hgb) values on hip fracture mortality [22,27,29]. The Hgb level of the patients at the time of admission was determined as the third parameter in our scoring system. Albumin is a significant parameter that informs us about the metabolic status of the patients, and low levels of albumin affect mortality rates in hip fractures [19,30,31]. Therefore, our scoring system determined the albumin value as the fourth parameter. Based on the effects of creatinine values on hip fractures, which are an indicator of kidney functions, the creatinine levels of patients at the time of admission were determined as the fifth parameter in our scoring system [32]. Extensive research has been conducted on the possible relationship between preoperative the neutrophile-lymphocyte ratio (NLR) and mortality in hip fractures; this was revealed in a large-scale meta-analysis. Using these data from the literature, the sixth parameter in our scoring system is NLR [33]. The seventh and last parameter in our scoring system is the monocyte–lymphocyte ratio (MLR). The relationship between the different values of MLR and hip fracture mortality has also been assessed in various studies [34,35,36].

The mortality score that was used to determine the three-year mortality of hip fractures is given in Table 1.

A process for examining the conformity of continuous variables to the normal distribution of measurements is the calculation of skewness and kurtosis values. These values obtained from the measurements are considered sufficient for normal distribution to be between +3 and −3 [37]. Variables that do not provide normal distribution have been identified in the measurements. It is appropriate to use parametric tests for measurements that provide normal distribution, and nonparametric methods for measurements that do not.

Statistical analysis was performed using IBM SPSS Statistics version 27.0, with a 95% confidence level. Categorical variables were expressed as frequencies and percentages [n (%)], while continuous variables were summarized using mean ± standard deviation (SD), median, minimum, and maximum values. The normality of distribution was assessed using the Kolmogorov–Smirnov test. Parametric tests were applied for normally distributed data, and non-parametric tests were used otherwise. The diagnostic performance of the scoring system was evaluated using receiver operating characteristic (ROC) analysis. Sensitivity, specificity, positive predictive value (PPV), and negative predictive value (NPV) were calculated to assess the model’s ability to discriminate between survivors and non-survivors.

## 3. Results

Along with the inclusion and exclusion parameters, the data of 1075 patients were included in this study. A proportion of 46.3% of the patients in the study died; 52.8% of the patients had fractures on the right side and 47.2% on the left side; 67.4% of the fractures were intertrochanteric femur fractures (ITFFs), and 32.6% were femoral neck fractures (FNFs); 30.5% of the patients were between the ages of 65 and 74, 33.8% were between the ages of 75 and 84, and 35.7% were over 85. The largest age group consisted of patients over the age of 85. Women constituted the majority with 65.6%, men with 34.4%.

The relationship between mortality and side, fracture type, age group, sex, hemoglobin level, albumin level, creatinine level, NLR ratio and MLR ratio of the patients included in the study is shown in Table 2.

Although more women died in absolute numbers (311 females vs. 187 males), this was due to their greater representation in the study population (65.6%). When analyzed by sex-specific subgroups, the mortality rate was 50.5% in males and 44.1% in females, indicating that male patients had a relatively higher risk of mortality.

There is a statistically significant relationship between mortality and age, sex, hemoglobin level, albumin level, creatinine level, NLR ratio, and MLR ratio in patients. Regarding mortality, the rates for being 85 years of age or older (48.2%), being male (37.6%), having hemoglobin level over 12 (14.5%), having creatinine level > 1 (48.4%), having NLR ratio > 6.55 (54.2%), and having MLR ratio > 0.635 (25.1%) are higher. The relationship is not significant for side and type.

The age range is 65 to 102, with a median value of 81. The mean age is 80.24 ± 8.38. Hemoglobin levels range from 6 to 21.6, with a median value of 12. Albumin levels range from 21 to 52, with a median value of 40. The mean albumin level is 39.29 ± 4.40. Creatinine levels range from 0.29 to 2.00, with a median level of 0.90. The mean creatinine level is 0.95 ± 0.35. The neutrophil percentage ranges from 13 to 95, with a median value of 79. The mean neutrophil percentage is 76.85 ± 11.19. The neutrophil–lymphocyte ratio ranged from 0.24 to 47.50, with a median value of 6.67. The mean NLR value was 8.33 ± 6.38. The monocyte percentage ranged from 0.90 to 16, with a median value of 5. The mean monocyte percentage was 4.78 ± 1.86. The monocyte–lymphocyte ratio ranged from 0.01 to 2.67, with a median value of 0.38. The mean MLR value was 0.46 ± 0.31. The lymphocyte percentage ranged from 2 to 70, with a median value of 12. The mean lymphocyte percentage was 14.10 ± 8.84. The total score ranged from 7 to 16, with a median value of 12. The mean total score was 11.63 ± 1.58.

The mean age in the non-mortality group was 78.19 ± 8.15 years, while the mean in the mortality group was 82.62 ± 8.01 years (*p* = 0.000 < 0.05). The mortality group had a significantly higher age. The mean hgb level in the non-mortality group was 12.13 ± 1.76 g/dL, while the mean in the mortality group was 11.66 ± 2.00 g/dL (*p* = 0.000 < 0.05). The hgb levels in the non-mortality group were significantly higher. The mean albumin level in the non-mortality group was 40.06 ± 3.77 g/L, while the mean in the mortality group was 38.40 ± 4.90 g/L (*p* = 0.000 < 0.05). The albumin levels in the non-mortality group were significantly higher. In terms of creatinine, the non-mortality group average was 0.91 ± 0.32 mg/dL, while the mortality group average was 1.00 ± 0.37 mg/dL (*p* = 0.000 < 0.05). The mortality group creatinine levels were significantly higher. In terms of NLR (Neutrophil–Lymphocyte Ratio), the non-mortality group average was 7.73 ± 5.71, while the exit group average was 9.02 ± 7.02 (*p* = 0.006 < 0.05). The mortality group NLR was significantly higher. The non-mortality group monocytes average was 4.67 ± 1.73, while the mortality group average was 4.91 ± 1.99 (*p* = 0.033 < 0.05). The mortality group monocyte values were significantly higher. In terms of MLR (Monocyte–Lymphocyte Ratio), the non-mortality group mean is 0.42 ± 0.28, while the mortality group mean is 0.50 ± 0.34 (*p* = 0.000 < 0.05). The mortality group MLR is significantly higher. In terms of lymphocytes, the non-mortality group mean is 14.80 ± 9.07, while the mortality group mean is 13.29 ± 8.51 (*p* = 0.002 < 0.05). The non-mortality group lymphocyte values are significantly higher. In terms of Total Score, the non-mortality group mean is 11.20 ± 1.51, while the mortality group mean is 12.11 ± 1.52 (*p* = 0.000 < 0.05). The mortality group Total Score is significantly higher. There is no significant difference between the groups in terms of Neu (Neutrophil) (*p* > 0.05).

The correlation between the parameters is shown in Table 3.

There are significant relationships between Total Score and all other variables, especially positive correlations with Creatinine level (r = 0.572, *p* < 0.001), NLR ratio (r = 0.390, *p* < 0.001), MLR ratio (r = 0.321, *p* < 0.001), Hemoglobin level (r = 0.196, *p* < 0.001), Albumin level (r = 0.114, *p* < 0.001), Sex group (r = 0.327, *p* < 0.001), and Age group (r = 0.460, *p* < 0.001). It is seen that the variables used in the Total Score measurement do not have high correlations within themselves. This situation shows that the measurements will not create a problem due to high correlation within themselves. Each of the measurements can be used independently.

ROC analysis was used to examine the predictability of mortality with Total Score measurement. Correct prediction probabilities were calculated. In addition, when age, sex, Hgb level, albumin level, creatinine level, NLR ratio, and MLR ratio measurements were excluded, the ROC analysis results were examined separately. The data related to this are given in Table 4.

A binary logistic regression analysis was conducted to investigate the relationship between the Akcaalan Score and 3-year mortality. The analysis demonstrated that the score was a statistically significant predictor of mortality (*p* < 0.001). Specifically, each 1-point increase in the Akcaalan Score increased the odds of mortality by 30.9% (Odds Ratio: 1.309; 95% CI: 1.194–1.411). Additionally, based on ROC curve analysis, when the Akcaalan Score exceeds 11.5, the probability of mortality within the first three years after hip fracture was calculated as 63.9%. This result underscores the prognostic value of the score and its potential use in clinical decision-making for elderly patients with hip fractures.

Although there were partial differences, when the sub-calculation factors were separately removed from the Total Score, there was no obvious significant difference. Therefore, it was determined that it was important to use all the parameters in the calculation of the Total Score measurement. Although it was seen that the sensitivity measurements increased by removing some of the measurements, this did not create a parallel increase for specifity.

**Figure 1:**
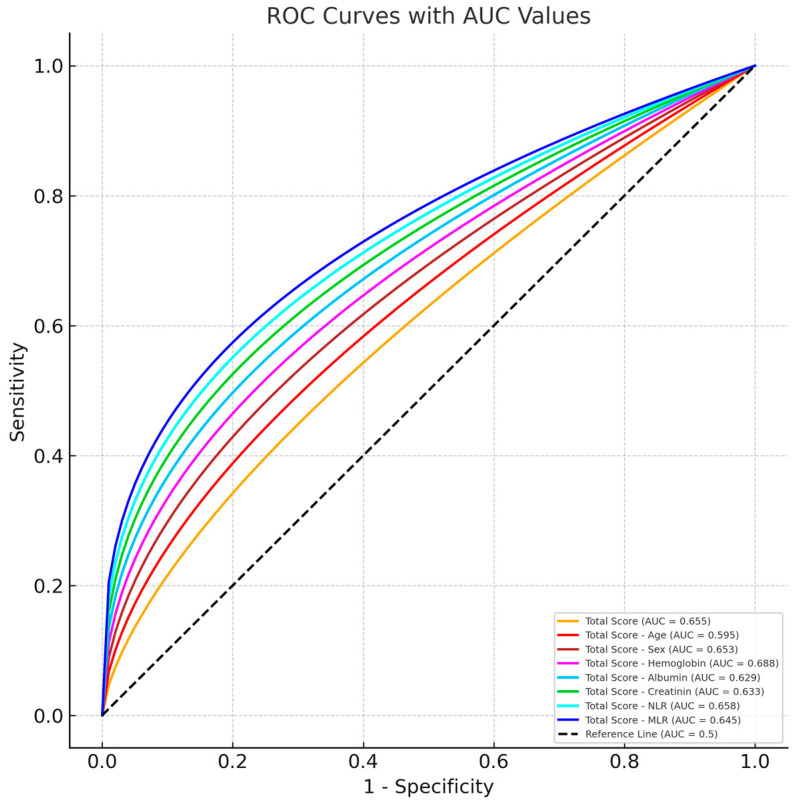
Graph of ROC Curve analysis for mortality score.

Total Score and ROC Curves formed when variables are removed from Total Score Calculation and Total Score ROC Curve are shown in Figure 1.

## 4. Discussion

The most significant finding in this study concerns the promising results of the mortality scores, which were generated using first-admission demographic data and the blood parameters of the patients who applied to the hospital for hip fracture to foresee mortality for patients in the postoperative three-year follow-up. Although parameters such as age, sex, and initial blood values cannot be modified at the time of admission, identifying patients at higher risk based on these parameters can assist clinicians in making more informed decisions regarding monitoring intensity, prioritization for surgery, or perioperative care strategies. Therefore, while the parameters themselves are fixed, the awareness of elevated risk may allow for more tailored management, potentially reducing preventable complications and improving outcomes.

Demographical characteristics, such as age and sex, can play a vital role in mortality for hip fractures. Yombi et al., in their study evaluating mortality in hip fractures in older people, showed that being male and at an advanced age increased hip fracture mortality [29]. In another study, Liu revealed that each one-year increase in age correspondingly increases mortality by 1.51-fold. Similarly, Vasoughi et al. showed that every one-year increase in age increases the mortality risk by 1.08-fold [23,24]. Extensive research has been conducted with different populations, and these studies also reveal similar effects of age and sex on mortality [22,38,39,40]. While earlier literature has reported male sex as a risk factor for increased mortality after hip fractures, our study initially appeared to contradict this due to the higher number of deceased female patients. However, when adjusted for group size, the mortality rate among males (50.5%) was indeed higher than among females (44.1%), which is consistent with existing findings in the literature. Parallel to studies in the literature, our scoring system reveals that age is associated with postoperative mortality in elderly hip fractures (*p* = 0.0001).

Anemia is prevalent in patients with hip fractures; while the Hgb value of 40.4% of these patients is under 12 g/dL, the Hgb value of the 12.3% portion is under 10 g/dL [41]. There is no determined cut-off value for Hgb values associated with mortality in hip fractures. Various studies have revealed different values. In a study in which 30-day and 180-day mortalities were evaluated among 1101 patients, the cut-off value was determined as 12 g/dL, and a significant correlation was found between the Hgb values and mortality [27]. In a study by Zhang, which included 2589 patients and evaluated 3-year mortality, the cut-off score was determined as 9.8 g/dL, and a relationship between Hgb values and mortality was shown [26]. Other research determined that the cut-off value was 10 g/dL at a preoperative low Hgb level [42,43]. In the present study, we categorized the Hgb value into three groups based on similar studies in the literature while determining the mortality score. A significant correlation was found between Hgb values and mortality (*p* = 0.0001).

Serum albumin levels are accepted as indicators of protein depletion and are frequently used in numerous metabolic evaluations. Generally, the serum albumin levels test is affordable and easily accessible. Low levels of albumin in patients with hip fractures are associated with morbidity and mortality rates [32,44]. However, there is no definitive number for the determined cut-off values. In another study, Harrison clearly showed that low values of albumin are associated with an increase in mortality in patients with hip fractures; the author found that average albumin levels were 29.5 g/dL in patients who expired and 32.8 g/dL in patients who survived [19]. Similarly, Wang and Pimlott determined the cut-off value for albumin as 35 g/dL, showing that low albumin values increased mortality in patients with hip fractures [30,31]. However, Özel et al. [45] retrospectively scanned 275 elderly patients with hip fractures and found the average albumin value among dead patients to be 30 (28–32)g/dL. In this study, we also detected a difference in albumin values between surviving and expired patients and categorized the findings under three different intervals in our scoring system.

Patients with higher plasma creatinine values are 2.5 times more likely to expire than individuals in a normal population [46]. Demirel et al., in their study in which 763 patients with hip fractures were retrospectively scanned and evaluated for three-month mortality, found the average creatinine levels in deceased patients to be 1.5 mg/dL and 0.97 mg/dL in surviving patients [47]. The meta-analysis conducted by Laulund et al. evaluated three different studies and revealed a relationship between creatinine levels and mortality [32]. In two of these studies, the findings showed a significant correlation between creatinine values and mortality; while one study determined the cut-off value as 1.768 mg/dL, the other resulted in a 1 mg/dL cut-off value [48,49]. The average creatinine value in patients who lost their lives in our study in the postoperative process was found to be 0.99 mg/dL, and the average creatinine value in surviving patients was 0.91 mg/dL. These results show that there is a significant difference between the two groups in terms of creatinine values.

NLR, a recently introduced inflammatory indicator, is the ratio of the absolute neutrophil count to the absolute lymphocyte count. This indicator is a sign of systemic inflammatory load. NLR is a relatively cheap and easily accessible routine laboratory indicator [50]. The systemic immune inflammation index is associated with reduced death rates for all reasons in elderly patients with hip fractures [51]. Chen et al., in their meta-analysis observing the relationship between NLR and mortality in hip fractures, included eight retrospective studies and 1563 patients; the authors revealed that high preoperative and postoperative NLR and high mortality rates were associated [33]. Different cut-off values for NLR were obtained in the studies in this meta-analysis. Bingöl et al. studied the 30-day and one-year mortality in their study with 345 patients, and the NLR cut-off value was 6.55 [36]. In their retrospective study with 1085 patients, Long et al. determined that the cut-off value for NLR was 7.25 [35]. However, the previously-mentioned meta-analysis also evaluated these two studies in terms of sensitivity and specificity, and Bingöl et al.’s study exhibited the highest sensitivity and specificity. Therefore, we determined the cut-off value for NLR to be 6.55 in our scoring system. We also found a statistically significant correlation between the two groups regarding NL values (0.0001). NLR, a recently introduced inflammatory indicator, was calculated in this study using the percentage values of neutrophils and lymphocytes, rather than their absolute counts.

Similar to NLR, MLR is also a relatively inexpensive and accessible parameter that can be easily obtained from routine blood parameters that show the inflammatory status of patients through systemic response. MLR has been associated with poor survival in elderly patients with hip fractures [34,36]. Long et al. showed a relationship between hip fractures and NLR in their study evaluating 1085 patients; the authors indicated a cut-off value of 0.76; however, the sensitivity and specificity were low [35]. While Bingöl et al. determined the cut-off value as 0.635, Tekin et al. indicated a cut-off value of 0.54. In this study, we found a statistically significant difference between both groups for NLR and determined the cut-off value as 0.635 in our scoring system. MLR is also a relatively inexpensive and accessible parameter that was calculated in this study using the percentage values of monocytes and lymphocytes, as an indicator of systemic inflammatory status.

Boukebous et al. published a study in which they evaluated 7756 hip fracture patients using the French national database; in this study, they developed a scoring system consisting of gender, age, the type of treatment (osteosynthesis or arthroplasty), and the Charlson score [52]. They showed that this scoring system could be a good clinical tool in predicting 90-day mortality and complications. By combining two different scoring systems created using different parameters, new developments may occur in the future that will provide more information on hip fracture mortality. However, unlike AtoG, the scoring system we created can also be used to decide on surgical timing, which is an important issue for the treatment of elderly hip fractures. In addition to its benefits in mortality prediction, the scoring system used for elderly hip fracture mortality can be used as an educational tool to increase the awareness of health professionals in the treatment of elderly hip fractures. Alain and his colleagues clearly demonstrated this in their study using a different scoring system [53].

The parameters included in our scoring system are not only statistically associated with mortality but also reflect underlying systemic conditions that contribute to poor clinical outcomes. Advanced age is commonly linked with frailty and reduced physiological reserve, making elderly patients more vulnerable to perioperative stress and complications [54]. Hypoalbuminemia is a marker of malnutrition and systemic inflammation, both of which have been associated with delayed wound healing, increased infection risk, and elevated postoperative mortality [20]. Similarly, elevated creatinine levels may indicate impaired renal function, which can compromise metabolic clearance and fluid–electrolyte balance, thereby increasing perioperative morbidity and mortality [14]. Furthermore, high neutrophil and monocyte counts relative to lymphocytes, expressed as elevated NLR and MLR values, are indicative of a pro-inflammatory state that predisposes patients to infections, delayed recovery, and multi-organ stress. These parameters, although not modifiable at admission, help identify high-risk patients and may guide clinicians in tailoring perioperative management to reduce avoidable complications.

The timing of surgery in elderly patients following a hip fracture is a controversial topic [55].While clinical guidelines commonly recommend surgical intervention within 24 to 48 h following hospital admission to reduce mortality and improve outcomes [53,56,57], this recommendation may not apply uniformly to all patients. In certain cases—especially among frail elderly patients with systemic instability, comorbidities, or abnormal laboratory values—rushing to surgery without adequate preoperative optimization may increase perioperative risk. In such instances, a short, well-planned delay may result in better outcomes. This view is supported by Greve et al., whose registry study of 59,675 Swedish patients found no significant association between surgical delay and mortality [58]. Similar findings were reported in their subsequent study in the Netherlands [59], highlighting that surgical timing should be individualized based on clinical context. In this regard, the mortality scoring system developed in our study may assist clinicians in stratifying patients upon admission. If a patient presents with a score above 11.5, this may warrant closer monitoring and consideration of medical optimization before surgery. However, since some parameters (e.g., age, sex, baseline lab values) are not modifiable, the scoring system should not be used to delay surgery indiscriminately, but rather to help inform perioperative decision-making and resource allocation.

This study has several limitations. First, its retrospective design and single-center setting may limit the generalizability of the findings. Second, patients with significant comorbid conditions (e.g., active malignancies, end-stage renal disease, hematological disorders) were excluded to reduce potential confounding effects. However, this selection may restrict the applicability of the scoring system to broader, real-world patient populations. Third, the scoring model was developed solely based on demographic and admission laboratory parameters. While these are easily obtainable and cost-effective, the exclusion of other known predictors, such as comorbidity indices (e.g., Charlson), pre-fracture functional status, surgical timing and details and postoperative complications, may have limited the model’s predictive capacity. Fourth, although the study included a 3-year follow-up period, it did not assess the impact of the scoring system on clinical decision-making, such as surgical timing or intervention strategies. Thus, the clinical utility of the score remains unproven. Fifth, external and prospective validation was not performed. Although the proposed score demonstrated statistical significance in this cohort, its sensitivity and specificity were only moderate. Further validation in larger, more diverse, and prospective multicenter cohorts is necessary before the score can be reliably adopted in clinical practice. Despite these limitations, one of the strengths of our study is that the parameters used in the scoring system are routinely available in clinical practice and require no additional cost, making it practical and feasible for real-time use [22,27].

## 5. Conclusions

The Akcaalan Mortality Score can provide satisfactory data to predict the three-year mortality of hip fractures in elderly patients during the preoperative process. The mortality score is >11.5, the probability of hip fracture mortality within the first three years is 63.9%. In addition to the mortality prediction, the mortality score can be used to assess surgical timing in these cases. Prospective studies are required to improve the mortality score. Identifying the preoperative mortality score can be useful as it positively affects prognosis through extensive evaluation and appropriate treatment methods.

## Figures and Tables

**Table 1:** Parameters, cut-off values and scoring system used in Akcaalan Mortality Score.

Parameters	Cut-Off Value	Score
Age	65–74 years	1
	75–84 years	2
	>85 years	3

Sex	Female	1
	Male	2

Hemoglobin Level	>12 mg/dL	1
	9.8–12 mg/dL	2
	< 9.8 mg/dL	3

Albumin Level	>35 g/L	1
	19.5–35 g/L	2
	<19.5 g/ L	3

Creatinin Level	<0.87 mg/dL	1
	0.87–1.2 mg/dL	2
	>1.2 mg/dL	3

NLR	<6.55	1
	>6.55	2

MLR	< 0.635	1
	>0.635	2
		
Total Score		7–18

**Table 2:** Data showing the relationship between clinical characteristics of patients and mortality.

		Mortality						*p*
		No (n = 577)		Yes (n = 498)		Total (n = 1075)		
		n	%	n	%	n	%	
Side	Right	295	51.1	273	54.8	568	52.8	0.226
	Left	282	48.9	225	45.2	507	47.2	
Fracture Type	CFF	188	32.7	162	32.6	350	32.6	0.972
	ITFF	387	67.3	335	67.4	722	67.4	
Age group	65–74	232	40.2	96	19.3	328	30.5	0.000 *
	75–84	201	34.8	162	32.5	363	33.8	
	85>	144	25.0	240	48.2	384	35.7	
Sex	Female	394	68.3	311	62.4	705	65.6	0.045 *
	Male	183	31.7	187	37.6	370	34.4	
Hemoglobin Level (mg/dL)	>12	44	7.6	72	14.5	116	10.8	0.000 *
	9.8–12	182	31.5	183	36.7	365	34.0	
	9.8<	351	60.8	243	48.8	594	55.3	
Albumin Level (g/L)	>35	540	93.6	409	82.1	949	88.3	0.000 *
	29.5–35	33	5.7	63	12.7	96	8.9	
	<29.5	4	0.7	26	5.2	30	2.8	
Creatinin Level (mg/dL)	<0.87	302	52.3	201	40.4	503	46.8	0.000 *
	0.87–1	78	13.5	56	11.2	134	12.5	
	>1	197	34.1	241	48.4	438	40.7	
NLR Ratio	<6.55	299	51.8	228	45.8	527	49.0	0.048 *
	>6.55	278	48.2	270	54.2	548	51.0	
MLR Ratio	<0.635	498	86.3	373	74.9	871	81.0	0.000 *
	>0.635	79	13.7	125	25.1	204	19.0	

* *p* < 0.05 significant relationship. *p* > 0.05 no significant relationship; Chi-square test CFF: Collum Femoris Fracture; ITFF: Intertrochanteric Femur Fracture.

**Table 3:** The relationships between age, sex, HGB level, albumin level, creatinine level, NLR ratio, MLR ratio and total score are shown.

		Age Group	Sex Group	Hemoglobin Level	Albumin Level	Creatinin Level	NLR Level	MLR Level	Total Score
Age group	rho	1	−0.136 **	−0.101 **	0.001	0.059	0.024	0.021	0.460 **
	*p*		0.000	0.001	0.974	0.051	0.437	0.488	0.000
Sex group	rho		1	0.080 **	0.014	0.089 **	0.052	0.014	0.327 **
	*p*			0.009	0.649	0.003	0.086	0.649	0.000
Hemoglobin level	rho			1	−0.307 **	−0.128 **	−0.007	−0.108 **	0.196 **
	*p*				0.000	0.000	0.813	0.000	0.000
Albumin levle	rho				1	−0.017	−0.003	0.073 *	0.114 **
	*p*					0.585	0.926	0.016	0.000
Creatinin level	rho					1	−0.060 *	−0.033	0.572 **
	*p*						0.050	0.275	0.000
NLR level	rho						1	0.384 **	0.390 **
	*p*							0.000	0.000
MLR level	rho							1	0.321 **
	*p*								**0.000**
Total Score	rho								1
	*p*								

** *p* < 0.01. * *p* < 0.05 significant relationship. *p* > 0.05 no significant relationship. 0 ≤ r ≤ 0.25 very weak. 0.26 ≤ r ≤ 0.49 weak. 0.50 ≤ r ≤ 0.69 moderate. 0.70 ≤ r ≤ 0.89 strong. 0.90 ≤ r ≤ 1 very strong; Spearman correlation test, rho (Spearman’s correlation coefficient).

**Table 4:** Predictability of mortality score measurement and ROC Analysis.

Scale	Area	*p*	95% CI		Cut Off Score	Sensitivity	Specificity	Positive Predictive Value	Negative Predictive Value
			Lower	Upper					
Total Score	0.655	0.000 *	0.623	0.687	11.5	0.649	0.568	0.652	0.565
Total Score - Age	0.595	0.000 *	0.561	0.629	9.5	0.582	0.549	0.604	0.527
Total Score - Sex	0.653	0.000 *	0.620	0.685	10.5	0.582	0.655	0.645	0.593
Total Score - Hemoglobin	0.688	0.000 *	0.656	0.719	8.5	0.789	0.461	0.717	0.558
Total Score - Albumin	0.629	0.000 *	0.596	0.662	9.5	0.793	0.345	0.659	0.511
Total Score - Creatinin	0.633	0.000 *	0.600	0.666	9.5	0.647	0.532	0.636	0.544
Total Score - NLR	0.658	0.000 *	0.626	0.691	9.5	0.769	0.452	0.694	0.548
Total Score - MLR	0.645	0.000 *	0.612	0.677	10.5	0.598	0.610	0.638	0.570

* *p* < 0.05 significant relationship. ROC (receiver operating characteristic).

## Data Availability

The data presented in this study are available on request from the corresponding author.
